# Indirect Effects of Oral Tolerance Inhibit Pulmonary Granulomas to *Schistosoma mansoni* Eggs

**DOI:** 10.1155/2012/293625

**Published:** 2011-10-13

**Authors:** Geraldo Magela Azevedo, Raquel Alves Costa, Mariana Araujo Resende, Claudiney Melquiades Rodrigues, Nelson Monteiro Vaz, Cláudia Rocha Carvalho

**Affiliations:** ^1^Departamento de Morfologia, ICB-UFMG, Avenue Antônio Carlos 6627, Pampulha, Belo Horizonte, MG, CEP 31270-901, Brazil; ^2^Departamentos de Bioquímica e Imunologia, Instituto de Ciências Biológicas, Universidade Federal de Minas Gerais, Belo Horizonte, MG, CEP 31270-901, Brazil

## Abstract

Parenteral injection of tolerated proteins into orally tolerant mice inhibits the initiation of immunological responses to unrelated proteins and blocks severe chronic inflammatory reactions of immunological origin, such as autoimmune reactions. This inhibitory effect which we have called “indirect effects of oral tolerance” is also known as “bystander suppression.” Herein, we show that i.p. injection of OVA + Al(OH)_3_ minutes before i.v. injection of *Schistosoma mansoni* eggs into OVA tolerant mice blocked the increase of pulmonary granulomas. In addition, the expression of ICAM-1 in lung parenchyma in areas outside the granulomas of OVA-orally tolerant mice was significantly reduced. However, at day 18 after granuloma induction there was no difference in immunofluorescency intensity to CD3, CD4, F4/80, and**α**-SMA per granuloma area of tolerant and control groups. Reduction of granulomas by reexposure to orally tolerated proteins was not correlated with a shift in Th-1/Th-2 cytokines in serum or lung tissue extract.

## 1. Introduction

Oral tolerance is a T-cell-mediated phenomenon described as the inhibition of immune responsiveness to a protein previously contacted by the oral route [1, 2]. Oral tolerance may prevent autoimmune and allergic diseases by mechanisms that are still controversial [3–5]. Two aspects of oral tolerance are of special interest to us because they may reflect its broad and systemic character, suggesting that more insights into these issues may improve our knowledge of the mechanisms of oral tolerance. First, oral tolerance is never absolute (complete), that is, parenteral immunization of tolerant animals with the tolerated antigen may induce antibody formation at levels inversely proportional to the ingested (tolerizing) dose of the antigen, but, this antibody formation can no longer be boostered by further parenteral immunizations [6]. Second, parenteral reexposure to a tolerated antigen blocks the initiation of immune responses to a second unrelated antigen—the effect we have named “indirect effect of oral tolerance” [7, 8] and is also known as “bystander suppression” [3, 9]. We have shown that such inhibitory effect occurs with different orally tolerated antigens and even when the tolerated antigen and the second unrelated antigen are injected into separated sites [7, 8]. The inhibitory indirect effects of oral tolerance does not require the simultaneous injection of the tolerated protein and the second antigen: it is still present 72 h after an injection of the tolerated antigen, but does not occur if the tolerated protein is injected after the second antigen [10]. Furthermore, parenteral re-exposure to a tolerated antigen has systemic effects on the migration of leucocytes and bone-marrow eosinopoiesis [11], blocks delayed-type hypersensitivity (DTH) reactions triggered by keyhole limpet haemocyanin (KLH) and paw oedema triggered by carragenan [12]. Amazingly, the indirect effects of oral tolerance to OVA also hinder the inflammation after an incisional skin lesion and improve wound healing in skin reducing fibrosis [13].

Granulomatous inflammation is involved in a number of diseases, and chronic granulomatous inflammation can cause damage and fibrosis to surrounding tissue [14, 15]. In schistosomiasis mansoni, the chronic egg-induced granulomatous response in the liver and intestines may eventually cause extensive tissue scarring and development of portal hypertension [16]. Immune responses to products secreted by the eggs (soluble egg antigens, SEA) result in the formation of granulomas that are composed of macrophages, eosinophils, lymphocytes, and fibroblasts [17]. Similarly to other inflammatory reactions, one critical aspect of granuloma formation is leukocyte migration dependent on the expression of adhesion molecules and cytokines [14, 18, 19]. Granulomatous inflammation triggered by *S. mansoni* eggs and the subsequent fibrosis has been considered a Th2-cytokine-driven inflammation [20]. However, different cytokines including IL-4, TNF-*α*, IL-10, and IFN-*γ* are produced during the course of granuloma formation [21]. *Schistosoma mansoni *eggs injected into the tail vein of mice are transported into the lung tissue via the pulmonary arteries where they become trapped within the lung parenchyma [22, 23]. The injection of *S. mansoni* eggs into normal mice allows the study of granulomatous reaction to the eggs without interference of additional factors triggered by the presence of the worms and reduces the variability in the size of granulomas otherwise produced by natural oviposition [21, 22]. Using the pulmonary granuloma model we have previously shown that indirect effects of oral tolerance triggered by i.p. injection of dinitrophenylated conjugates of OVA (DNP-OVA) emulsified in complete Freund's adjuvant (CFA) inhibit the formation of pulmonary granulomas [24].

To further characterize the indirect effects of oral tolerance upon inflammatory reactions, we tested if re-exposure of OVA orally tolerant mice to OVA + Al(OH)_3_ block the concomitant formation of pulmonary granuloma. Mice orally tolerant to OVA and controls not tolerant were i.p. injected with OVA concomitant with i.v. injection of* S. mansoni *live eggs. We compared granulomas size from day 1 to day 18 after i.v. eggs, granuloma cellular composition, spleen, lung and serum cytokines levels, and the expression of intercellular adhesion molecule-1 (ICAM-1) in the lung.

## 2. Materials and Methods

### 2.1. Animals

8-week-old female C57BL/6 mice were bred and maintained in the animal breeding unit at the Institute of Biological Sciences, Universidade Federal de Minas Gerais (UFMG), Brazil. The animals were fed, housed, and treated according to the guidelines of the Ethics Committee of Animal Experimentation (CETEA) of the UFMG. Experimental groups contained at least five mice per each time point.

### 2.2. Feeding Regimens for Oral Tolerance Induction

Oral tolerance to ovalbumin (OVA) was induced by requiring mice to drink, *ad libitum*, a 1 : 5 solution of chicken egg white in drinking water for 3 consecutive days. The egg white solution was prepared in our laboratory from commercially available eggs and contained an average of 4 mg OVA/mL. Daily estimated average consumption was 20 mg OVA/mouse, and this resulted in significant levels of tolerance [25]. Bottles were changed every day to avoid contamination. Control groups received filtered tap water. Oral treatment was discontinued 7 days before granuloma induction.

### 2.3. Pulmonary Granuloma

7 days after oral tolerance induction, control and experimental animals were injected i.v. with 2,000 eggs from *S. mansoni* through a tail vein. Live *S. mansoni* eggs were purified from the livers of *S. mansoni* cercariae-infected Swiss mice, which were kindly provided by Dr. Débora Negrão Correa, from Universidade Federal de Minas Gerais, Brasil.

### 2.4. Parenteral Immunizations

Purified OVA was obtained commercially (grade V, Sigma, St. Louis, MO). Mice which had been pretreated orally with egg white (tolerant group) and control mice (immune group) received one intraperitoneal (i.p.) injection of 0.25 mL of a suspension containing 10 *μ*g OVA plus 1.6 mg Al(OH)_3_ immediately before the i.v. egg injection. The other control group (granuloma group) was not i.p. immunized.

### 2.5. Bleeding

Blood samples were collected in the absence of anticoagulant, and serum samples were obtained and stored at −20°C until used in a serum antibody assay to test for tolerance induction or cytometric bead array (CBA) for quantitative analysis of cytokines.

### 2.6. Sacrifice

Mice were sacrificed by cervical dislocation 1, 5, 11, 14, and 18 days after inoculation of *S. mansoni* eggs; lungs were collected and fixed for either histology or immunostaining. In one experiment the spleens were also collected.

### 2.7. Histology

For histology lungs were fixed immediately in Carson's modified Millonig's phosphate buffered formalin (pH = 7,0 for 24 h) and embedded in paraffin. Serial sections of 4 *μ*m were stained with hematoxylin and eosin (HE) or Gomori's trichrome for bright field microscopy. Digital images of tissues were obtained using a BX50 Olympus microscope (Olympus, Japan) and an Olympus Q Colour 3 Camera, which was connected to a computer running the Q-Capture Pro software program (Q Imaging, Canada).

### 2.8. Morphometry

The areas of the granulomas were measured in a blinded fashion on digitalized photomicrographs of HE-stained sections with Image Tool 3.0 software (UTHSCSA, San Antonio, Tex, USA http://ddsdx.uthscsa.edu/dig/itdesc.html).

### 2.9. Immunostaining and Confocal Microscopy

Immunofluorescence labeling and quantitative confocal microscopy were used to investigate the distribution and quantity of macrophages (F4/80), lymphocytes (CD3+), CD4+ cells, myofibroblast (*α*-SMA), and ICAM-1. Briefly, lungs were immediately fixed and cryosubstituted in a −80°C solution containing 80% methanol and 20% dimethyl sulfoxide for 5–7 days, transferred to −20°C for 1-2 days, and then brought to room temperature as described elsewhere [26]. Samples were rinsed 3X in absolute ethanol, 2X in xylene and embedded in paraplast following standard protocols. Five *μ*m longitudinal sections from the middle of the lung were dewaxed with xylene and rehydrated through a graded series of ethanol into PBS. Blocking was achieved using 2% BSA in PBS at room temperature for 1 h followed by an overnight incubation at 4°C with primary antibodies diluted in PBS containing 0.1% BSA and 0.01% Tween-20. The following antibodies were used: rat anti-F4/80 (eBioscience San Diego, CA), rat anti-CD3 (Pharmigen, San Diego, CA), rat anti-CD4 (Pharmigen, San Diego, CA), mouse anti-*α*-SMA (Sigma St. Louis, MO) and mouse anti-ICAM-1 (R&D Systems, San Diego, CA). After 4-5 rinses in PBS, sections were incubated for 1 h at room temperature in the dark with Alexa 488-conjugated goat antimouse IgG secondary antibodies (Molecular Probes, Eugene, OR) or FITC-conjugated goat antirat IgG-polyclonal secondary antibody (eBioscience San Diego, CA). Nuclear counterstainig was made with 4′6-diamidino-2-phenylindol (DAPI). After several rinses in PBS, sections were mounted in a mixture of 10% 1.0 M Tris-HCl, pH 9.0, and 90% glycerol and analyzed using a laser scanning confocal microscope (Zeiss 510META; Carl Zeiss AG, Oberkochen, Germany). Optimal confocal settings (aperture, gain, and laser power) for each antibody used were determined at the beginning of each imaging session and then held constant during the analysis of all the samples.

The distribution patterns and levels of expression of F4/80, CD3, CD4, *α*-SMA, and ICAM-1 were analyzed on digitalized photomicrographs with Image Tool 3.0 software (UTHSCSA, San Antonio, Tex, USA, http://ddsdx.uthscsa.edu/dig/itdesc.html). Images were captured at 12 bit and analyzed in the gray scale range of 0 to 255. Green fluorescence intensity was recorded as the sum of gray values of all pixels divided by the area (in *μ*m^2^) ×10^−3^. Background fluorescence was measured in each sample and subtracted from the values obtained for the fluorescence intensity.

### 2.10. Spleen Cell Cultures and Cytokine Assay

Spleen cells were counted and adjusted to concentrations of 1 × 10^7^ cells/mL in RPMI 1640 supplemented with 2% heat inactivated FCS, 2 mM L-glutamine (Sigma-Aldrich, Inc.), and antibiotics (100 U/mL penicillin, 100 lg/mL streptomycin) (Sigma-Aldrich, Inc.). Cells were cultured in 96-well flat-bottom plates at 125 *μ*L/well in a humidified atmosphere with 5% CO_2_ with or without soluble schistosome egg antigens at 50 *μ*g/mL culture fluid, ovalbumin at 1 mg/mL, or concanavalin A at 2 *μ*g/mL. After 72 h, supernatant fluids were harvested and frozen −20°C for subsequent cytokine analysis. The production of IL-10 and IFN-*γ* by spleen cells was measured by cytokine capture ELISA.

### 2.11. Quantitative Analysis of Serum and Lung Cytokines

Serum samples were collected as previously described and stored at −20°C until used. One hundred milligrams of lung tissue samples from animals of each experimental groups were homogenized in 1 mL of PBS (0.4 M NaCl and 10 mM de NaPO4) containing proteases inhibitors (0.1 mM phenylmethylsulfonyl fluoride, 0.1 mM benzethonium chloride, 10 mM EDTA, and 20 KI aprotinin A) and 0.05% Tween 20. The samples were then centrifuged for 10 minutes at 3,000 ×g and the supernatant immediately used for quantitative analysis of cytokines. The cytokines (IL-2, IL-4, IL-5, IFN-*γ*, and TNF-*α*) in serum and lung samples were measured with Cytometric Bead Array (CBA) Mouse Th1/Th2 kit according to the manufacturer's specifications (BD Biosciences, CA, USA).

### 2.12. Antibody Assay

Anti-OVA and antisoluble egg antigen (SEA) antibody titres were determined by standard enzyme-linked immunosorbent assay (ELISA) using an automatic ELISA reader (BioRad, Hercules, CA). ELISA scores were computed by calculating the sums of the optical densities obtained from the six serum dilutions between 1 : 50 and 1 : 1600 of individual mice. The details of the assay method have been described previously [11, 24, 27]. Each score shown represents the mean ± SEM of the 5 animals in the group.

Statistical Analysis was performed using GraphPad Prism 4 (GraphPad Software, CA, USA), and the statistical significance of differences between groups was determined using one-way ANOVA followed by Student-Newman-Keuls test. Values of *P* ≤ 0.05 were considered significant. The results are expressed as the mean ± SEM.

## 3. Results

### 3.1. Reexposure of OVA-Orally Tolerant Animals to the Tolerated Antigen in Al(OH)_3_ Blocks Granuloma but Not Anti-SEA Antibody Formation

**Figure 1:**
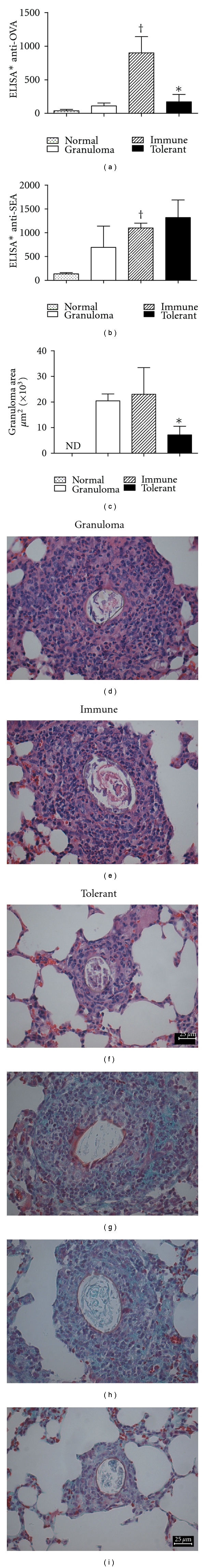
Reduction of granuloma by re-exposure of orally tolerant animals to the tolerated antigen. Serum levels of (a) anti-OVA antibodies and (b) anti-SEA antibodies and (c) pulmonary granuloma area and (d–i) histological aspect of pulmonary granuloma 18 days after i.v. injection of *S. mansoni* eggs in nonimmunized mice (granuloma group, open bars), OVA immune controls (hatched bars), and OVA-orally tolerant (black bars). Normal mice (doted bars) were not immunized with OVA neither injected with eggs. Data represent mean ± SEM. *P ≤ 0.05 tolerant versus immune ^†^ *P* ≤ 0.05 immune versus normal. nd: not detected. Original magnification of HE (d–f) and Gomori's trichrome (g–i) photomicrographs 400X; scale bars = 25 *μ*m.

To induce oral tolerance to OVA C57BL/6 mice were offered an egg white solution for three days as their only liquid source (called “tolerant”), and control mice (called “immune”) drank tap water. Seven days after interrupting the oral treatment, mice were immunized i.p. with OVA in Al(OH)_3_ immediately before the i.v. injection of live *S. mansoni* eggs. Another control group (called “granuloma”) received i.v. injection of eggs without any other previous treatment. Eighteen days thereafter, mice were sacrificed and blood and lung were removed for serum antibodies and pulmonary granuloma evaluation.  Figure 1(a) shows that the oral pretreatment with egg white resulted in tolerance to OVA, that is, anti-OVA antibodies were significantly inhibited as compared with immune mice not orally pretreated. In contrast, anti-SEA antibodies were augmented in all groups injected with live eggs, irrespective of other treatments (Figure 1(b)). Noteworthy, granuloma area was significantly smaller in OVA-tolerant mice (Figure 1(c)). 

We also performed histological analyses of Gomori's trichrome (Figures 1(g)–1(i)) and HE-stained lung sections (Figures 1(d)–1(f)). Eighteen days after i.v. injection of eggs, pulmonary granulomas were well organized with concentric arrangement and composed of macrophages, eosinophils, lymphocytes and some fibroblasts and epithelioid cells (Figures 1(d)–1(f)). Granulomas were observed around small branches of pulmonary arteries. Initial collagen deposition could be better observed after staining with Gomori's trichrome in all groups (Figures 1(g)–1(i)). Inflammatory infiltrates in lung parenchyma and alveolar macrophages were characteristic of all groups, but more prominently in immune group. Of note, in OVA-tolerant mice the majority of eggs were surrounded by typical, although small, granulomas (Figure 1(f)) and their lung parenchyma presented less inflammatory infiltrates (not shown).

### 3.2. Re-Exposure of OVA Orally Tolerant Animals to the Tolerated Antigen Blocks Initial Phases of Pulmonary Granuloma Formation

**Figure 2:**
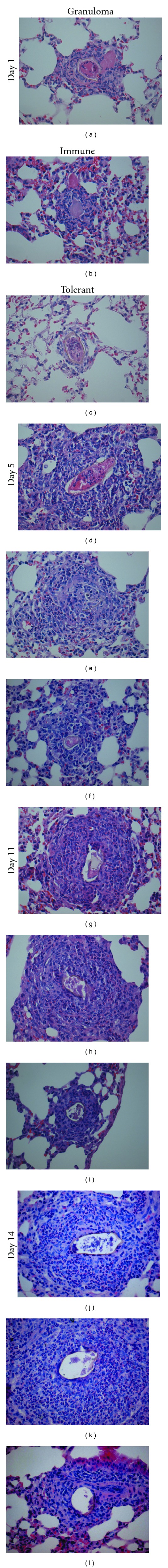
Granuloma at different times after egg injection. Lung HE staining 1, 5, 11, and 14 days after i.v. injection of *S. mansoni* eggs. (a–c) At day 1, an inflammatory infiltrate with predominance of neutrophils and macrophages can be detected around eggs in all groups, but it is less intense in the tolerant group. (d–f) At day 5, macrophages, eosinophils, and some lymphocytes can be detected. (g–l) At days 11 and 14 granulomas are more organized and some fibroblasts can be detected. Granulomas in tolerant mice follow the same pattern of organization but do not reach the same size of granulomas in controls group. Scale bars = 25 *μ*m.

As described in the literature, the granuloma formed around *S. mansoni* eggs has a defined maturational stage followed by a stage of involution, and, from a morphological point of view, these stages may be classified as pregranulomatous and granulomatous stages [28]. The pregranulomatous, exudative stage is characterized by accumulation of neutrophils, eosinophils, and macrophages around the egg. The granulomatous stage can be divided into three phases: exudative-productive, productive, and involutional. In order to compare the kinetics of granuloma formation in OVA tolerant and not tolerant mice we performed histological analyses of Gomori's trichrome (not shown) and HE-stained lung sections.  Figure 2 shows HE-stained sections of the pregranulomatous stage at days 1 and 5 after egg inoculation (Figures 2(a)–2(f)) and granulomas in the exudative-productive phase of the granulomatous stage at days 11 and 14 (Figures 2(g)–2(l)). At day 14, scarce deposition of collagen fibres could be observed in Gomori's trichrome-stained sections (not shown). Granulomas in the tolerant group followed similar kinetics as that from controls group, but less intense.

**Figure 3:**
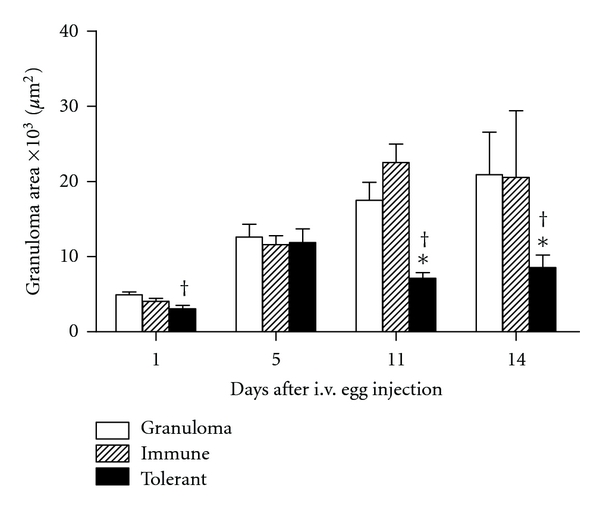
Re-exposure of orally tolerant animals to the tolerated antigen block enlargement of granuloma area. The area of granulomas at days 1, 5, 11 and 14 after i.v. injection of eggs in nonimmunized mice (granuloma group, open bars), OVA immune controls (hatched bars), and OVA-orally tolerant (black bars). Data represent mean ± SEM (five mice/group). *P ≤ 0.05 tolerant versus immune and ^†^ *P* ≤ 0.05 tolerant versus granuloma.

Morphometric analysis (Figure 3) showed that at day 1 the area of granulomas from OVA-tolerant mice is reduced as compared to control mice, but this difference disappears at day 5. However, after day 5, the area of granulomas increased in controls group and became significantly higher than the area of granulomas in OVA-tolerant mice at days 11 and 14 (Figure 3).

### 3.3. Re-Exposure of Orally Tolerant Mice to the Tolerated Antigen Do Not Change Granuloma Cell Composition

**Figure 4:**
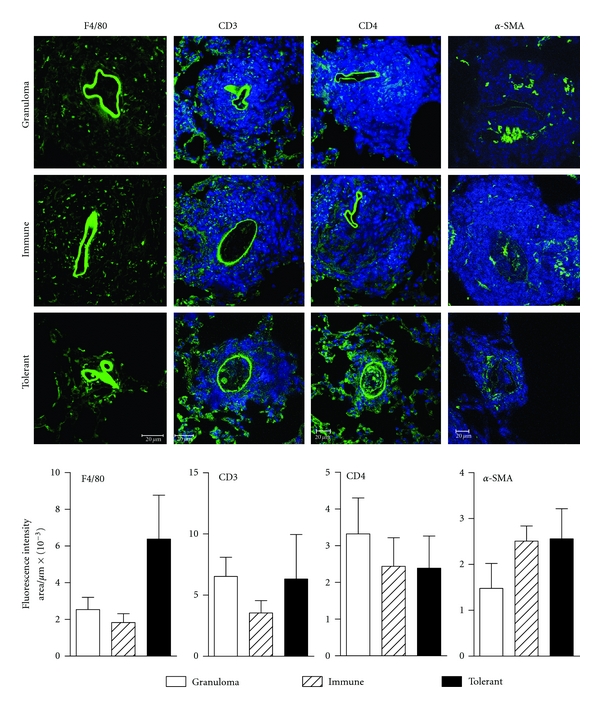
Cell subsets in pulmonary granulomas 18 days after i.v. egg injection. Immunolocalization using specific antibodies followed by secondary antibodies coupled with fluorescein (green) and nuclear counterstainig with 4′6-diamidino-2-phenylindol (blue), 18 days after i.v. eggs injection. Confocal microscope images were captured with a 63X objective, and the graphs represent the green fluorescence intensity (the sum of gray values of all pixels divided by the area (in *μ*m^2^) × 10^−3^) of expression of F4/80 (macrophages), CD3 (T-lymphocytes), CD4+ cells, and *α*-SMA (myofibroblasts) in nonimmunized mice (granuloma group, open bars), OVA immune controls (hatched bars), and OVA-orally tolerant (black bars). Data represent mean ± SEM of fluorescence intensity of duplicate slides (*n* = 5 mice/group). The green autofluorescency of eggs was excluded from all analyses. No significant difference was found between groups.

To further characterize granuloma cell composition macrophages, T lymphocytes and myofibroblasts were identified and quantified by immunostaining followed by confocal microscopy. Despite their smaller area, granulomas from tolerant mice present the same cell subsets as the large granulomas of controls groups (Figure 4). Even myofibroblasts (*α*-SMA+) were present in the smaller granulomas of the Ova orally-tolerant mice (Figure 4). For technical reasons we could not perform double immunostaining with anti-CD3 and anti-CD4 antibodies. To quantify fluorescency, images were captured at 12 bit and analyzed in the gray scale range of 0 to 255. Green fluorescence intensity was recorded as the sum of gray values of all pixels divided by the area (in *μ*m^2^) ×10^−3^ as described in Section 2. The green auto-fluorescency of eggs was excluded from all analyses. No significant difference in fluorescency intensity was found between groups. Then we can conclude that the reduction in the area of granulomas is due to proportional reduction of the inflammatory cells.

### 3.4. Re-Exposure of Orally Tolerant Mice to the Tolerated Antigen Reduces ICAM-1 Expression

**Figure 5:**
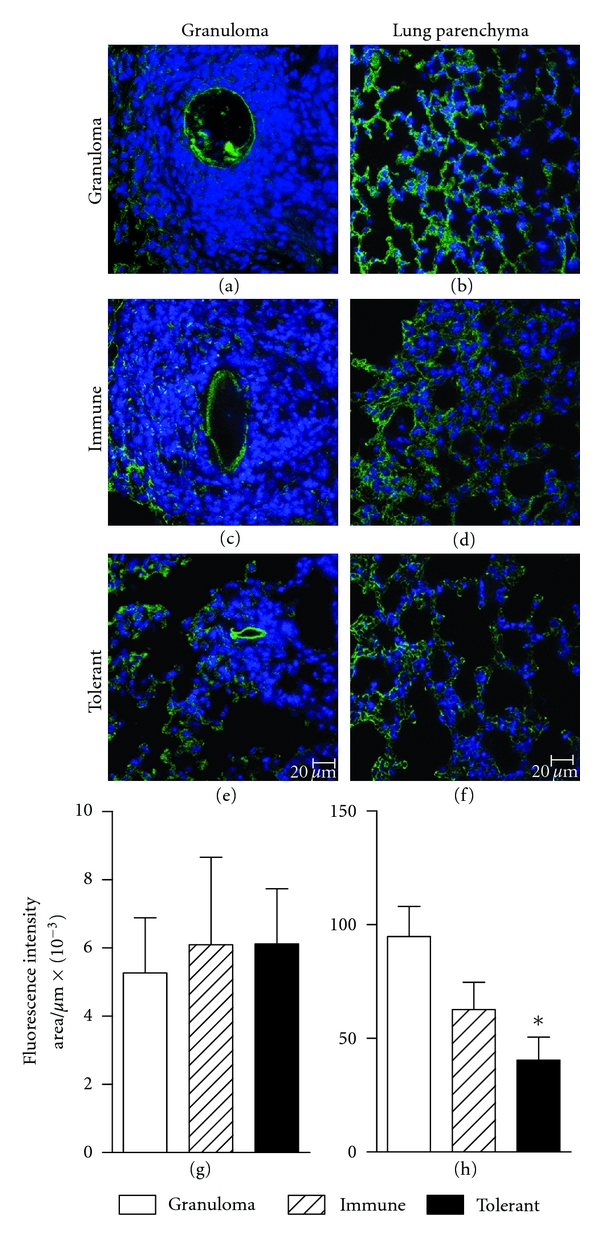
Re-exposure of orally tolerant animals to the tolerated antigen block the rise of ICAM-1 expression in lung parenchyma. Immunolocalization of ICAM-1 in granulomas and lung parenchyma using specific antibody coupled with fluorescein (green) and nuclear counterstainig with 4′6-diamidino-2-phenylindol (blue), 18 days after i.v. eggs injection. Confocal microscope images were captured with a 63X objective, and the graphs represent the green fluorescence intensity (the sum of gray values of all pixels divided by the area (in *μ*m^2^) × 10^−3^) of expression of ICAM-1 in the granuloma area (a–d) and in lung parenchyma (e–h) in nonimmunized mice (granuloma group, open bars), OVA immune controls (hatched bars), and OVA-orally tolerant mice (black bars). Data represent mean ± SEM of fluorescence intensity of duplicate slides (*n* = 5 mice/group).

Adhesion molecules enable circulating leukocytes to accumulate in areas of lung inflammation, and adhesion is the initial phase of a process whereby activated endothelial cells induce leukocyte migration into tissues. As ICAM-1 has been described as a predominant adhesion molecule after egg deposition in the liver of *S. mansoni* infected mice [19] we compared its expression in lungs after i.v. injection of eggs. Our results show that the majority of *S. mansoni* egg-induced ICAM-1 expression 18 days after pulmonary granuloma induction was restricted to the lung parenchyma outside the granulomas (Figure 5). The intensity of ICAM-1 expression in the granuloma of tolerant and controls group was not different. However, in the lung parenchyma outside granulomas, the expression of ICAM-1 was significantly inhibited in the tolerant mice (Figure 5).

### 3.5. Reduction of Granulomas by Re-Exposure to Orally Tolerant Proteins Was Not Correlated with a Shift in Th-1/Th-2 Cytokines

**Figure 6:**
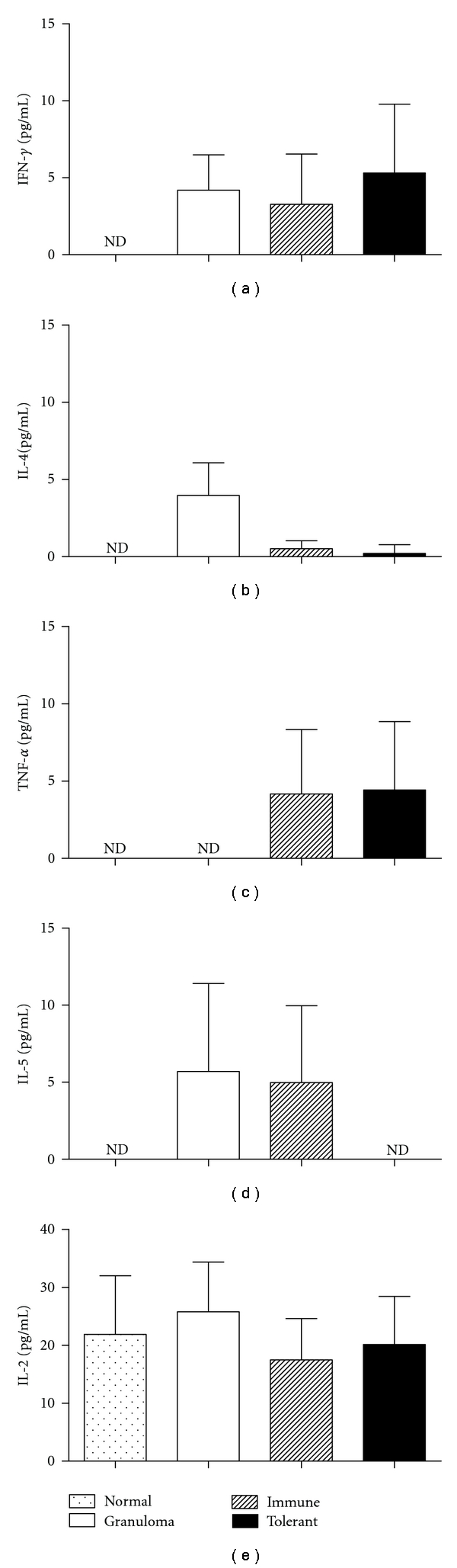
Cytokines production of lungs. Fourteen days after i.v. injection of *S. mansoni* eggs in nonimmunized mice (granuloma group, open bars), OVA immune controls (hatched bars), and OVA-orally tolerant mice (black bars), lungs were removed and homogenized in extract buffer. Normal mice (doted bars) were not immunized with OVA and neither injected with eggs. Extract supernatant was collected for cytokine assay. IFN-*γ*, TNF-*α*, IL-2, IL-4, and IL-5 were measured using a Cytometric Bead Array (CBA) kit. The results are shown as mean concentrations ± SEM. nd: not detected.

**Figure 7:**
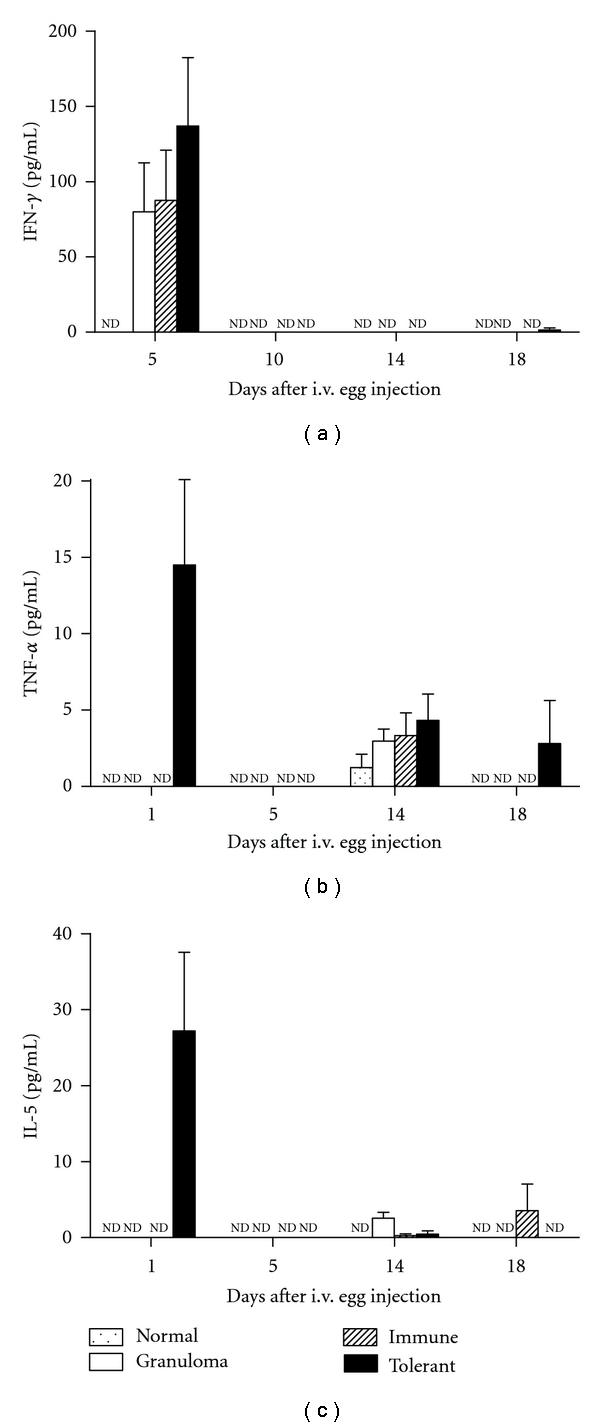
Time course of serum cytokines after granuloma induction. IFN-*γ*, TNF-*α*, IL-2, IL-4, and IL-5 were measured using a Cytometric Bead Array (CBA) kit in serum samples collected from nonimmunized mice (granuloma group, open bars), OVA immune controls (hatched bars), and OVA-orally tolerant mice (black bars). Normal mice (doted bars) were not immunized with OVA and neither injected with eggs. The results are shown as mean concentrations ± SEM. nd: not detected. IL-2 and IL-4 were not detected.

Using a commercial kit to detect typical Th1/Th2 cytokines, we compared the levels of IFN-*γ*, TNF-*α*, IL-2, IL-4, and IL-5 in lung homogenates 14 days after granuloma induction (Figure 6) and in serum samples (Figure 7) collected 1, 5, 14 and 18 days after granuloma induction. IFN-*γ* could be detected in lung homogenates (Figure 6) and serum at day 1 (Figure 7) in some mice injected with *S. mansoni* eggs and not in normal (naïve) mice, but no difference was found between the experimental groups. IL-2 and IL-4 were not detected in serum samples from any group. IL-2 was detected in the same level in lung homogenates of normal and experimental groups, and the low levels of IL-4 detected in lung homogenates of immune and tolerant mice did not correlate with the size of their granulomas. TNF-*α* was detected in the serum at the same level in all groups, 14 days after egg injection. At day 1, TNF-*α*, and IL-5 were detected in the serum only in the tolerant group, but not in all mice from this group. In conclusion, reduction of granulomas in tolerant mice does not correlate with a shift in Th-1/Th-2 cytokines.

**Figure 8:**
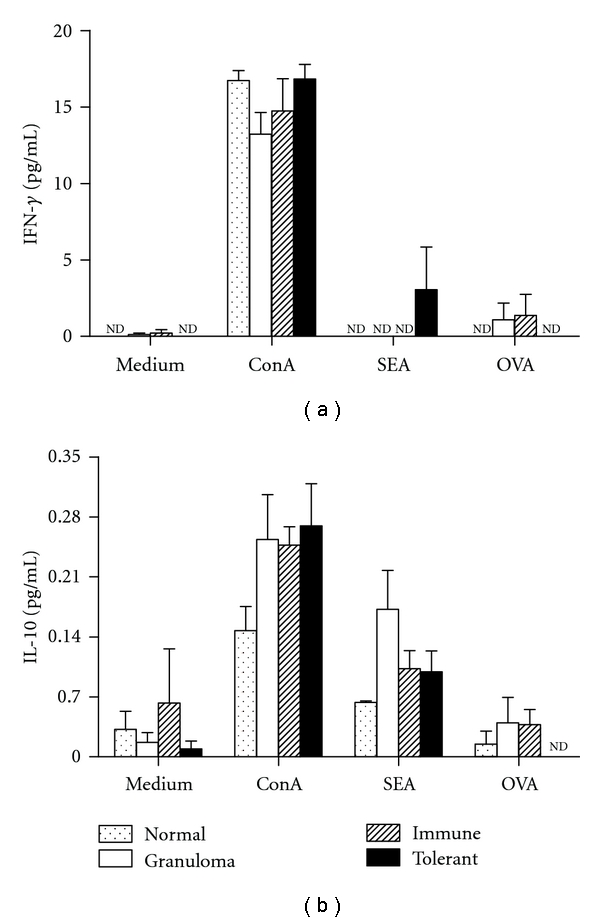
IFN-*γ* and IL-10 production of spleen cells stimulated with SEA or OVA. Eighteen days after i.v. injection of *S. mansoni* eggs in nonimmunized mice (granuloma group, open bars), OVA immune controls (hatched bars), and OVA-orally tolerant mice (black bars) spleen cells were cultured with medium, ConA, SEA or OVA for 3 days. Normal mice (doted bars) were not immunized with OVA neither injected with eggs. The culture supernatant fluids were harvested and IFN-*γ* and IL-10 measured by sandwich ELISA. The results are shown as mean concentrations ± SEM. nd: not detected.

We also compared the production of IFN-*γ* and IL-10 by spleen cell restimulated “in vitro” with OVA or SEA. The results in Figure 8 do not make us confident to attribute the reduction of granulomas in tolerant mice to systemic alteration in the production of these cytokines.

## 4. Discussion

The standard protocols used to demonstrate tolerance in orally pre-treated animals involve challenge with the antigen in adjuvant, and there is evidence that adjuvants play a significant role in tolerogenesis during the triggering/parenteral phase affecting the kind of Ig isotype that is suppressed or maintained for long periods after oral feeding [29, 30]. Tobagus et al. [30] suggested that when a Th1-selective adjuvant (such as CFA) is used the resulting response displayed selective inhibition of the Th1 component (IFN-*γ*) of the immune response while orally pre-treated animals challenged with the antigen in a Th2-selective adjuvant (alum) displayed a selective inhibition of Th2 responses. While oral tolerance is specific to the antigen contacted by the oral route, it is noteworthy that the parenteral injection of small doses (e.g., 10 *μ*g) of proteins to which the animal is orally tolerant triggers a strong inhibition of primary responses to unrelated antigens [3, 8, 10].

In previous work we have shown that in mice orally-tolerant to ovalbumin (OVA), anti-SEA and pulmonary granulomas triggered by i.v. injection of eggs from *S. mansoni* were inhibited by i.p. injection of dinitrophenylated conjugates of OVA (DNP-OVA) emulsified in complete Freund's adjuvant (CFA) [24]. In that work we analysed granulomas only at day 18 after i.v. egg injection and found that the more prominent granulomas occurred in nontolerant mice concomitantly injected with DNP-OVA + CFA and small granulomas were found in orally tolerant mice injected with DNP-OVA + CFA. In the tolerant group eggs were predominant in intravascular locations with initial periovular reactions containing monocytes, eosinophils, and collagen fibers derived from the vascular wall [24]. Herein, we have shown that i.p. injection of OVA plus Al(OH)_3_ into OVA-tolerant mice also inhibited pulmonary granuloma but not anti-SEA antibodies production (Figure 1). Our previous report and the present one as well show that reduction of granuloma in orally-tolerant mice is independent of the kind of adjuvant used. On the other hand, inhibition of anti-SEA antibody formation only occurred when the tolerated antigens were injected with CFA [24]. Nevertheless, we have shown that inhibition of antibodies to other proteins such as KLH and haemoglobin occurs with injection of OVA + Al(OH)_3_ in OVA orally-tolerant mice [10, 12]. So, unknown factors associated with the eggs make it more difficult to inhibit the anti-SEA antibody response.

This and already published work [3, 9, 11–13] show that the re-exposure of orally tolerant animals to the tolerated antigen blocks inflammatory reactions. One hallmark of inflammatory processes is the migration of leukocytes to local areas. Herein we have shown that the injection of tolerated antigen into orally tolerant mice weakens the influx of leucocytes into the lung and reduces the size of granuloma (Figures 1, 2, and 3). However, the inhibitory effect of oral tolerance hindered the intensity of migration of cells into the lung, but not its kinetics, since granulomas followed the same pattern of formation in tolerant and not tolerant mice (Figure 2). Furthermore granulomas in tolerant mice have the same cell composition although in low numbers as compared to not tolerant mice (Figure 4).

Changes in the expression of cell adhesion molecules initiate leukocyte trafficking, and ICAM-1 is the predominant adhesion molecule in schistosome egg granuloma formation [19]. The reduction in the expression of ICAM-1 in tolerant mice, as shown herein (Figure 5), is certainly involved in the demonstrated inhibitory effect. We could not find significant changes in cytokine secretion, neither in the blood, nor in lung extracts (Figures 6 and 7). IL-10 was detected after spleen cell cultures with SEA, but no difference was found between tolerant and not tolerant group, and IL-10 concentrations in supernatants of spleen cells cultured with OVA were not different from basal production (Figure 8). This detection may require proper timing, but the present results argue against major changes in the Th1/Th2 axis.

It is important to pursue these findings with additional experiments. Antibody formation may be involved in the reduction of granulomas in orally-tolerant mice, since B cells and anti-idyotipic antibodies are involved in the regulation of granulomas [31, 32] and oral tolerance also affects B cell and antibody production [29]. In searching for possible mechanisms involved in inhibitory indirect effects triggered by parenteral injection of tolerated antigens we must keep in mind that they affect the initial phases of the inflammatory response which are thought to be primarily innate, as shown herein and in previous work [13]. This may be taken as indication that, in addition to specific immunological (clonal) events, the exposure to tolerated antigens triggers other phenomena, for example, of neuroendocrine nature.

## 5. Conclusion

Parenteral injection of tolerated proteins into orally tolerant mice blocked the increase of pulmonary granulomas and the expression of ICAM-1 in lung parenchyma in areas outside the granulomas. The reduction in the area of granulomas in tolerant mice is due to proportional reduction of the inflammatory cells and was not correlated with a shift in Th-1/Th-2 cytokines in serum or lung tissue extract.

## References

[1] Faria AM, Weiner HL (2006). Oral tolerance: therapeutic implications for autoimmune diseases. *Clinical and Developmental Immunology*.

[2] Vaz N, Faria AM, Verdolin BA, Carvalho CR (1997). Immaturity, ageing and oral tolerance. *Scandinavian Journal of Immunology*.

[3] Miller A, Lider O, Weiner H (1991). Antigen-driven bystander suppression after oral administration of antigens. *Journal of Experimental Medicine*.

[4] Russo M, Jancar S, Pereira de Siqueira AL (1998). Prevention of lung eosinophilic inflammation by oral tolerance. *Immunology Letters*.

[5] Yoshino S, Quattrocchi E, Weiner HL (1995). Suppression of antigen-induced arthritis in Lewis rats by oral administration of type II collagen. *Arthritis and Rheumatism*.

[6] Verdolin BA, Ficker SM, Faria AM, Vaz NM, Carvalho CR (2001). Stabilization of serum antibody responses triggered by initial mucosal contact with the antigen independently of oral tolerance induction. *Brazilian Journal of Medical and Biological Research*.

[7] Carvalho CR, Vaz NM (1996). Indirect effects are independent of the way of tolerance induction. *Scandinavian Journal of Immunology*.

[8] Carvalho CR, Verdolin BA, de Souza AV, Vaz NM (1994). Indirect effects of oral tolerance in mice. *Scandinavian Journal of Immunology*.

[9] Backstrom NF, Dahlgren UI (2004). Bystander suppression of collagen-induced arthritis in mice fed ovalbumin. *Arthritis Research & Therapy*.

[10] Carvalho CR, Verdolin BA, Vaz NM (1997). Indirect effects of oral tolerance cannot be ascribed to bystander suppression. *Scandinavian Journal of Immunology*.

[11] Rodrigues CM, Martins-Filho OA, Vaz NM, Carvalho CR (2006). Systemic effects of oral tolerance on inflammation: mobilization of lymphocytes and bone marrow eosinopoiesis. *Immunology*.

[12] Ramos G, Rodrigues CM, Azevedo GM, Pinho V, Carvalho CR, Vaz NM (2009). Cell-mediated immune response to unrelated proteins and unspecific inflammation blocked by orally tolerated proteins. *Immunology*.

[13] Costa RA, Ruiz-de-Souza V, Azevedo GM (2011). Indirect effects of oral tolerance improve wound healing in skin. *Wound Repair and Regeneration*.

[14] Gonzalez A, Lenzi HL, Motta EM (2005). Expression of adhesion molecules in lungs of mice infected with Paracoccidioides brasiliensis conidia. *Microbes and Infection*.

[15] Wilson MS, Mentink-Kane MM, Pesce JT, Ramalingam TR, Thompson R, Wynn TA (2007). Immunopathology of schistosomiasis. *Immunology and Cell Biology*.

[16] Abath FG, Morais CN, Montenegro CE, Wynn TA, Montenegro SM (2006). Immunopathogenic mechanisms in schistosomiasis: what can be learnt from human studies?. *Trends in Parasitology*.

[17] Co DO, Hogan LH, Il-Kim S, Sandor M (2004). T cell contributions to the different phases of granuloma formation. *Immunology Letters*.

[18] Pesce JT, Ramalingam TR, Wilson MS (2009). Retnla (relm*α*/Fizz1) suppresses helminth-induced Th2- type immunity. *Plos Pathogens*.

[19] Ritter DM, McKerrow JH (1996). Intercellular adhesion molecule 1 is the major adhesion molecule expressed during schistosome granuloma formation. *Infection and Immunity*.

[20] Schramm G, Haas H (2010). Th2 immune response against Schistosoma mansoni infection. *Microbes and Infection*.

[21] Chensue SW, Terebuh PD, Warmington KS (1992). Role of IL-4 and IFN-*γ* in Schistosoma mansoni egg-induced hypersensitivity granuloma formation: orchestration, relative contribution, and relationship to macrophage function. *Journal of Immunology*.

[22] Cheever AW, Lenzi JA, Lenzi HL, Andrade ZA (2002). Experimental models of Schistosoma mansoni infection. *Memorias do Instituto Oswaldo Cruz*.

[23] Nair MG, Du Y, Perrigoue JG (2009). Alternatively activated macrophage-derived RELM-*α* is a negative regulator of type 2 inflammation in the lung. *Journal of Experimental Medicine*.

[24] Carvalho CR, Lenzi HL, Correa-Oliveira R, Vaz NM (2002). Indirect effects of oral tolerance to ovalbumin interfere with the immune responses triggered by Schistosoma mansoni eggs. *Brazilian Journal of Medical and Biological Research*.

[25] Faria AM, Garcia G, Rios MJC, Michalaros CL, Vaz NM (1993). Decrease in susceptibility to oral tolerance induction and occurrence of oral immunization to ovalbumin in 20-38-week-old mice. The effect of interval between oral exposure and rate of antigen intake in the oral immunization. *Immunology*.

[26] Carvalhaes LS, Gervasio OL, Guatimosim C (2006). Collagen XVIII/endostatin is associated with the epithelial-mesenchymal transformation in the atrioventricular valves during cardiac development. *Developmental Dynamics*.

[27] Cunha AP, Oliveira RP, Junior AB, Vaz NM, Carvalho CR (2009). Different requirements for the adoptive transfer of oral tolerance and its indirect effects assessed by DTH and antibody responses in mice. *Cellular Immunology*.

[28] Lenzi HL, Kimmel E, Schechtman H (1998). Histoarchitecture of schistosomal granuloma development and involution: morphogenetic and biomechanical approaches. *Memorias do Instituto Oswaldo Cruz*.

[29] de Faria AM, Ficker SM, Speziali E (1998). Aging affects oral tolerance induction but not its maintenance in mice. *Mechanisms of Ageing and Development*.

[30] Tobagus IT, Thomas WR, Holt PG (2004). Adjuvant costimulation during secondary antigen challenge directs qualitative aspects of oral tolerance Induction, particularly during the neonatal period. *Journal of Immunology*.

[31] Jankovic D, Cheever AW, Kullberg MC (1998). CD4+ T cell-mediated granulomatous pathology in schistosomiasis is downregulated by a B cell-dependent mechanism requiring Fc receptor signaling. *Journal of Experimental Medicine*.

[32] Montesano MA, Colley DG, Willard MT, Freeman GL, Secor WE (2002). Idiotypes expressed early in experimental Schistosoma mansoni infections predict clinical outcomes of chronic disease. *Journal of Experimental Medicine*.

